# Supplementary figures and images for: Genome-Wide Identification and Transcriptional Expression of the METTL21C Gene Family in Chicken
Source: Genes (Basel). 2019 Aug 20;10(8):628. doi: 10.3390/genes10080628 (PMC6723737; doi:10.3390/genes10080628)

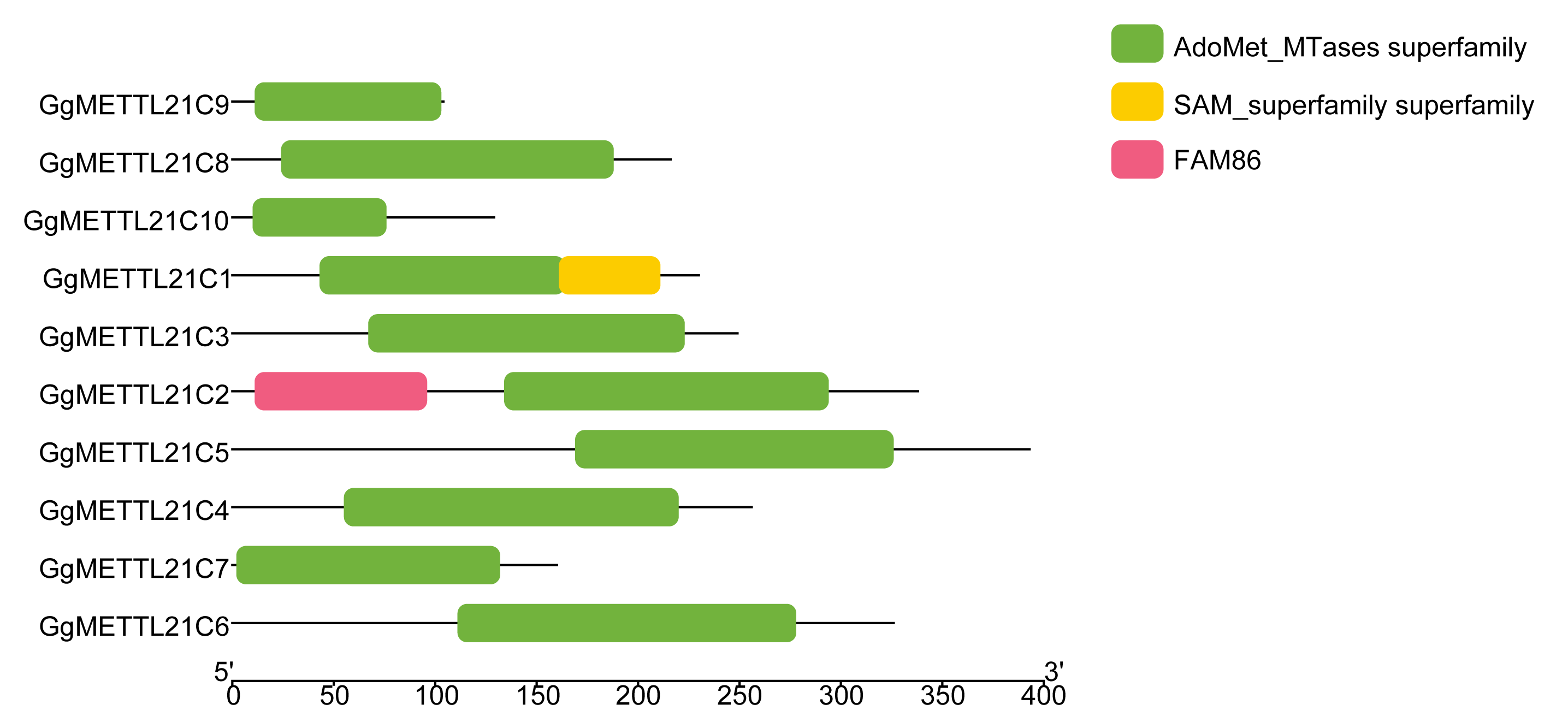

Supplement: Supplementary file 1 [file genes-10-00628-s001.zip › Supplementary Files/FigureS1.tif]
